# Androgen Deprivation Induces Reprogramming of Prostate Cancer Cells to Stem-Like Cells

**DOI:** 10.3390/cells9061441

**Published:** 2020-06-10

**Authors:** Belén G. Sánchez, Alicia Bort, Diana Vara-Ciruelos, Inés Díaz-Laviada

**Affiliations:** 1 Department of System Biology, Biochemistry and Molecular Biology Unit, School of Medicine and Health Sciences, University of Alcalá, 28871 Alcalá de Henares, Madrid, Spain; belen.sanchezg@edu.uah.es (B.G.S.); alicia.bort@edu.uah.es (A.B.); dianavara83@hotmail.com (D.V.-C.); 2 Chemical Research Institute “Andrés M. del Río” (IQAR), Alcalá University, 28871 Alcalá de Henares, Madrid, Spain

**Keywords:** AMPK, neuroendocrine cells, cancer stem cells, lineage plasticity, prostate cancer

## Abstract

In the past few years, cell plasticity has emerged as a mode of targeted therapy evasion in prostate adenocarcinoma. When exposed to anticancer therapies, tumor cells may switch into a different histological subtype, such as the neuroendocrine phenotype which is associated with treatment failure and a poor prognosis. In this study, we demonstrated that long-term androgen signal depletion of prostate LNCaP cells induced a neuroendocrine phenotype followed by re-differentiation towards a “stem-like” state. LNCaP cells incubated for 30 days in charcoal-stripped medium or with the androgen receptor antagonist 2-hydroxyflutamide developed neuroendocrine morphology and increased the expression of the neuroendocrine markers βIII-tubulin and neuron specific enolase (NSE). When cells were incubated for 90 days in androgen-depleted medium, they grew as floating spheres and had enhanced expression of the stem cell markers CD133, ALDH1A1, and the transporter ABCB1A. Additionally, the pluripotent transcription factors Nanog and Oct4 and the angiogenic factor VEGF were up-regulated while the expression of E-cadherin was inhibited. Cell viability revealed that those cells were resistant to docetaxel and 2-hidroxyflutamide. Mechanistically, androgen depletion induced the decrease in AMP-activated kinase (AMPK) expression and activation and stabilization of the hypoxia-inducible factor HIF-1α. Overexpression of AMPK in the stem-like cells decreased the expression of stem markers as well as that of HIF-1α and VEGF while it restored the levels of E-cadherin and PGC-1α. Most importantly, docetaxel sensitivity was restored in stem-like AMPK-transfected cells. Our model provides a new regulatory mechanism of prostate cancer plasticity through AMPK that is worth exploring.

## 1. Introduction

Prostate cancer (PC) is the most common malignancy in men and accounts for the second leading cause of cancer-related deaths in men in the developed world [1]. Patients that are not cured with surgery or radiation undergo androgen deprivation by chirurgical or endocrine therapy. Although this androgen deprivation therapy (ADT) is initially effective in suppressing tumor growth, most patients inevitably develop resistance to androgen deprivation therapy and experience cancer recurrence in an aggressive form named Castration Resistant Prostate Cancer (CRPC) of a very poor prognosis [2]. Currently, development of chemoresistance represents one of the greatest challenges in cancer treatment. Despite intense research, the mechanisms contributing to acquired resistance are not completely understood.

Recent theories postulate that as tumor cells display a high plasticity, therapy exposure and environmental signals can induce phenotype switching towards different phenotypes that are associated with drug resistance, a phenomenon named “lineage plasticity” [3,4]. For instance, in 25% of metastatic prostate carcinoma patients treated with anti-androgen therapy, tumor relapses with morphological features of neuroendocrine carcinoma named neuroendocrine prostate cancer (NEPC) [5,6]. It is worthy to note that neuroendocrine differentiation is associated with treatment failure and poor outcome in metastatic prostate cancer [5,7]. Moreover, ADT was shown to be a key driver of the neuroendocrine differentiation of the prostate adenocarcinoma to NEPC [8], and so it is not surprising that the incidence of treatment-induced NEPC is rising because of the widespread use of more potent androgen receptor (AR) pathway inhibitors [9]. These latter tumor types are refractory to hormonal therapies and are thought to represent a resistance driven by cellular lineage plasticity [3].

Neuroendocrine-like cells secrete peptide hormones and growth factors to support the growth of surrounding tumor cells in a paracrine manner and express high levels of survival genes, conferring resistance to treatments [6]. In addition, neuroendocrine prostate tumors are positive for neuroendocrine markers like neuron specific enolase (NSE), beta-III-tubulin, chromogranin A, or synaptophysin. Another typical modification in neuroendocrine derivatives of adenocarcinomas is the downregulation of androgen receptor, which indicates a reprogramming towards alternative pathways cooperating with the acquired resistance of NEPC. 

Other mechanisms of lineage plasticity involve the acquisition of undifferentiated features and stemness properties by adenocarcinoma cells, which can be a pathway of escape from suppressive anticancer therapy. Unlike the bulk of tumor cells, stem-like cells are refractory to traditional therapy and are probably responsible for relapse or disease recurrence in cancer patients. Cancer stem cells (CSCs) develop mechanisms of drug resistance like a high expression of membrane-bound efflux pumps, especially ATP-binding cassette (ABC) transporters such as ABCB1 (also known as MDR1 or P-glycoprotein) and detoxifying enzymes like the aldehyde dehydrogenase ALDH1A1, a cytosolic enzyme that affords protection from the adverse effects of chemotherapeutic insult. In addition, CSCs increase the expression of transcription factors involved in pluripotency and cell fate like Sox2, the octamer-binding protein 4 (Oct4), and Nanog, which confers the ability of reprogramming and plasticity. 

Thus, understanding the molecular mechanisms of these cellular rewiring processes involved in the acquisition of drug resistance might help to better design new therapeutic strategies. In recent years, research has been focused on phosphorylation as a tight regulator of the network of kinases responsible for the induction and maintenance of pluripotency [10]. Several protein kinases have been shown to regulate the pluripotency including the MAPK/Erk signal transduction cascade and the phosphoinositide 3-kinase (PI3K)/AKT serine threonine kinase signaling pathway. However, although AMP-activated kinase (AMPK) plays an essential role in cellular process involved in cell fate, such as ATP generation and autophagy, its role in reprogramming has been scarcely studied. Recent findings by Bandow et al. showed that AMPK is a negative regulator of osteogenic [11] and chondrogenic differentiation [12] and that AMPK activation by metformin inhibited the expression of Sox6 and Sox9 differentiation transcription factors [12]. The role of AMPK on cell differentiation has been reinforced by data showing that AMPK activation promoted monocyte-to-macrophage differentiation [13]. Interestingly, Vazquez-Martín et al. have demonstrated that pharmacological activation of AMPK impeded the reprogramming of mouse embryonic and human diploid fibroblasts into stem cells [14] and that acquired resistance to the AMPK activator metformin triggers a transcriptome reprogramming toward a metastatic stem-like profile [15]. We have recently shown that AMPK overexpression decreases stem features and transcription factors in hepatocellular carcinoma. Altogether, these data suggest that AMPK has a role in cell fate beyond its involvement in metabolic networks. 

In the present study, we induced reprogramming of LNCaP cells into cancer stem-like cells by long-term incubation with androgen deprivation. The cell lines generated were resistant to docetaxel and 2-hydroxyflutamide and expressed high levels of stem cell markers. Here, we show that AMPK is inhibited upon early cancer stem-like cell differentiation and that AMPK overexpression decreases neuroendocrine and stemness markers. These results suggest that AMPK is a new target to impact cell lineage plasticity. Additionally, the generation of resistant cell lines will allow for the study of mechanisms contributing to acquisition of the chemoresistance phenotype.

## 2. Materials and Methods

### 2.1. Materials

Dextran-coated charcoal and 2-hydroxyflutamide were purchased from Sigma-Aldrich (St. Louis, MO, USA). Docetaxel (DTX) was purchased from TOCRIS (Bristol, UK). All other chemicals were purchased from Sigma-Aldrich (St. Louis, MO, USA).

### 2.2. Cell Culture

The human prostate cancer cell line LNCaP was obtained from American Type Culture Collection (ATCC CRL-1740, Rockville, MD, USA) and cultured in RPMI-1640/10%FBS supplemented with 100 IU/mL penicillin G sodium, 100 g/mL streptomycin sulfate, and 0.25 g/mL amphotericin B (Invitrogen, Paisley, UK). Cancer stem-like cell lines were generated by long-term androgen ablation by two strategies. First, steroid removal was carried out by incubation of FBS with dextran-coated charcoal at 4 °C overnight and then centrifuged. LNCaP cells were maintained in RPMI-1640/10% charcoal stripped-FBS for three months and re-named LN-NE. On the other hand, LNCaP cells were cultured for two months in RPMI-1640/10% FBS with 1 μM 2-hydroxyflutamide and re-named LN-FLU. In addition, we used the LNCaP-abl cell line, a commercially available androgen-independent cell line, directly derived from LNCaP cells by maintenance in an androgen-deprived medium [16]. For treatment experiments, cells were plated and grown for 24 h. The medium was then replaced with serum-free RPMI 1640 and incubated with the different treatments for the indicated times.

### 2.3. Cell Proliferation Assay

Cell proliferation was analyzed using the MTT assay. Cells (1.5 × 10^5^cells/well) were seeded into 12-well plates and allowed to attach and grow for 24 h. After different treatments for 24 h, 100 μL of MTT (3-(4,5-dimethyl-2-thiazolyl)-2,5-diphenyl-2H-tetrazolium bromide) dye solution (Sigma-Aldrich) was added to each well and incubated at 37 °C for 1 h. Subsequently, the cells were lysed with 2-propanol to dissolve the formazan crystals. Then, the optical density of each well was measured using a microplate reader (iMARK, Bio-Rad Laboratories, Inc., Hercules, CA, USA) at a wavelength of 595 nm, and the nonspecific absorbance measured at 650 nm was subtracted. Cell viability was calculated as the percentage compared to the control cells, which were arbitrarily assigned 100% viability. 

### 2.4. Redifferentiation Assay

For the in vitro redifferentiation of stem-like cells into osteoblasts, stem-like cells (1 × 10^5^ cells/well) were seeded into 6-well plates and incubated in phenol red-free DMEM low-glucose medium (Invitrogen) supplemented with 2% CSS, 10 mM β-glycerophosphate, 50 μg/mL ascorbic acid-2-phosphate, 10 nM dexamethasone, and 10 nM 1,25-dihydroxyvitamin D_3_ for 15 days. For glial redifferentiation, stem-like cells were incubated in phenol-red free DMEM (Sigma-Aldrich, St. Louis, MO, USA) with 1% N-2 supplement (Invitrogen), 2% CSS, and 2 mM L-glutamine (Invitrogen Carlsbad, CA, USA).

### 2.5. Western Blot

Proteins for Western blotting were isolated by lysing cells in lysis buffer (50 mM Tris pH 7.4, 0.8 M NaCl, 5 mM MgCl_2_, 0.1% Triton X-100) containing protease inhibitor and phosphatase inhibitor cocktail (Roche, Diagnostics; Mannheim, Germany), incubated on ice for 15 min, and cleared by microcentrifugation. Twenty micrograms of total protein/lane was separated by SDS-polyacrylamide gel electrophoresis (SDS-PAGE) and then transferred onto a PVDF membrane. Membranes were incubated overnight at 4 °C with the primary antibodies. After washing in TTBS, membranes were incubated with peroxidase-conjugated anti-mouse or anti-rabbit secondary antibodies for 2 h at room temperature. The immune complex was visualized with an ECL system (Cell Signaling Technology, Danvers, MA, USA). Protein expression levels were quantified using Scion Image 4.0 (Scion Corporation, Chicago, IL, USA), normalized relative to the indicated housekeeping protein, and expressed as fold changes relative to the control treatment. Primary antibodies anti-PSMA (#12815, working dilution 1:1000), anti-CD133 (#64326, working dilution 1:500), anti-ALDH1A1 (#12035, working dilution 1:500), anti-p-AMPKα1-thr172 (#2531, working dilution 1:1000), p-ACC-ser79 (#3661, working dilution 1:1000), anti-PGC-1α, anti HIF-1α (#36169, working dilution 1:1000), anti E-cadherin (#3195, working dilution 1:500), and the antibodies against the corresponding total forms were obtained from Cell Signaling Technology (Danvers, MA, USA). Anti-βIII-tubulin (PRB-435P, working dilution 1:1000) was purchased from Covance (NJ, USA), anti-NSE (m0873, working dilution 1:500) from DAKO (Denmark), anti-AR (sc-7305, working dilution 1:500) from Santa Cruz Biotechnology (TX, USA), anti-GFAP (PA5-16291, working dilution 1:1000) and anti-VEGF (PA5-16754, working dilution 1:2000) were from Invitrogen (Carlsbad, CA, USA), and anti-Osx (#3195 working dilution 1:1000) was from Abcam (Cambridge, UK). Peroxidase-labeled secondary anti-mouse IgG (A9044, working dilution 1:5000) was from Sigma-Aldrich (St. Louis, MO, USA), and anti-rabbit IgG (#7074, working dilution 1:1000) was from Cell Signaling Technology (Danvers, MA, USA). 

### 2.6. RNA Extraction and Reverse Transcription Quantitative Polymerase Chain Reaction

Total cellular RNA was extracted from cells using the RNeasy Mini Kit (Qiagen, Hilden, Germany) according to the manufacturer’s protocol. Total RNA (2 μg) underwent cDNA synthesis using SuperScriptTM RT (Roche, Basel, Switzerland) according to the manufacturer’s protocol. qPCR was performed in a 10 μL volume using SYBR-Green PCR Master Mix (Takara Bio, Inc., Kusatsu, Japan) on a 7500 Real-Time PCR System (Applied Biosystems Inc., Foster City, CA, USA) according to the manufacturer’s protocols. PCR amplification was carried out using the following primer sequences: Nanog-F 5’-TTTGTGGGCCTGAAGAAAC-3’, Nanog-R 5’-AGGGCTGTCCTGAATAAGCAG-3’; Oct4-F 5’-GACAGGGGGAGGGGAGGAGCTAGG-3’, Oct4-R 5’-CTTCCCTCCAACCAGTTGCCCCAAAC-3’; ABCB1A-F 5’-TTGCTGCTTACATTCAGGTTTCA-3’, ABCB1A-R 5’-AGCCTATCTCCTGTCGCATTA-3’; OSX-F 5′-AGCGACCACTTGAGCAAACAT-3′, OSX-R 5′-GCGGCTGATTGGCTTCTTCT-3′; IBSP-F 5′-AAAGTGAGAACGGGGAACCT-3′, IBSP-R 5′-GATGCAAAGCCAGAATGGAT-3′.

### 2.7. siRNA Transfections

Cells were transfected in 1 mL OPTIMEM containing 4 μg Lipofectamine iMax (Invitrogen) with 100 nM AMPK-specific small interfering RNA (siRNA) duplexes (5′-CCCAUAUUAUUUGCGUGUAdTdT-3′ and 5′-UACACGCCAAAUAAUAUGGGdTdT-3′) (Ambion-Life Technologies, Carlsbad, CA, USA) or scrambled RNA (control) according to the manufacturer’s protocols (Invitrogen). At 48 h after transfection, the medium was removed and replaced with RPMI. At the indicated time points after transfection, cells were used for MTT cell viability assays or Western blot analysis.

### 2.8. Transient Transfections

LNCaP and LN-NE cells were co-transfected with 4 μg recombinant α1 (pcDNA5-FRT α1-Flag), β1 (pCMV β1-untagged), and γ1 WT (pcDNA5-Flpln-T10 γ1 WT-Flag) plasmids, kindly provided by Dr. Hardie (University of Dundee, UK), using 5 μL Lipofectamine 3000 (Invitrogen). After 48 h of transfection, the cells were collected and protein expression assayed by Western blotting. Anti-FLAG antibody (F3165, working dilution 1:5000) was obtained from Sigma-Aldrich.

### 2.9. Confocal Microscopy

The cells were fixed in 4% paraformaldehyde in PBS and incubated with 0.1% Triton X-100 for permeabilization. Immunolabelling with an anti-AMPK antibody (Cell Signaling Technology, Danvers, MA, USA) was performed by incubation at room temperature for 1 h. Secondary labeling was performed with Alexa Fluor 488-conjugated secondary antibodies (Invitrogen, Carlsbad, CA, USA). Coverslips were then mounted with DAPI-containing Mowiol mounting medium (Sigma-Aldrich, St. Louis, MO, USA). Imaging was performed with a Leica TCS SP5 laser-scanning confocal microscope with LAS-AF imaging software using a 40× oil objective. 

### 2.10. Statistical Analysis

Statistical significance was estimated with Graphpad 6.0 (La Jolla, CA, USA) software using 2-way ANOVA and Tukey’s or Sidak’s multiple comparisons test when indicated. Data are presented as the mean ± SD.

## 3. Results

### 3.1. Establishment of Prostate Stem-Like Cells

**Figure 1:**
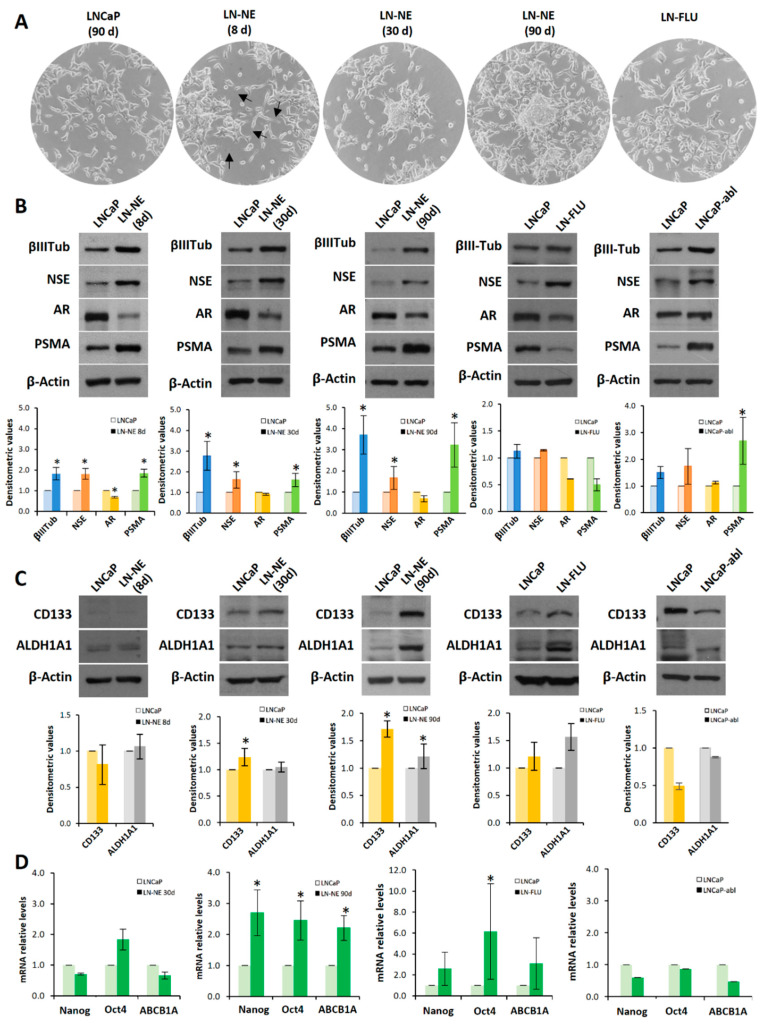
Long-term androgen withdrawal induces differentiation to prostate stem-like cells. (**A**) LNCaP and LN-NE cells were maintained in medium with or without androgens respectively. LN-FLU cells were adapted to grow in the presence of 2-hydroxyflutamide (FLU) for 2 months. The morphological appearance of the LN-NE cell line was observed by phase contrast microscopy at three different times: 8, 30, and 90 days, and that of the LN-FLU cell line at two months. Arrows point to neurites in LN-NE 8d cells. (**B**) Levels of expression of NED markers were determined by Western blot and limited to actin as a load control. The cell line LNCaP-abl was used for comparison. A representative image of three experiments is shown. (**C**) Levels of expression of CSC markers were determined by Western blot. Densitometric analysis (n = 3) is shown below each image. (**D**) Levels of expression of cancers stem cell (CSC) markers determined by qPCR. The data show the relative mRNA expression to GAPDH, which was used as a housekeeping gene. Data represent the mean ± SD of three independent experiments. * *p* < 0.05 significant difference between LNCaP and LN-NE cells or LNCaP and LN-FLU cells by two-way ANOVA and Sidak’s multiple comparisons test.

Prostate cancer cell lines can be used to investigate mechanisms of androgen deprivation resistance. To generate cancer models of therapy resistance, we produced a cell line adapted to grow in the absence of androgens. To that end, LNCaP cells were cultured in medium containing charcoal stripped-FBS for three months. Under this condition, LNCaP cells underwent a morphological change, and 8 days later, the appearance of many features of NE cells, like neurite outgrowth (cell body prolongation longer than twice the cell body diameter) could be observed (Figure 1A). 

Prolonged androgen deprivation (30 days) induced the condensation of cell body, the loss of cell–cell contacts and aggregation of cells, which resembled the features of neural stem cells [17,18]. When cells were grown with androgen withdrawal for three months, aggregates of cells adopted spheroid growth (Figure 1A), grew floating, and exhibited a slow dividing rate (not shown). Then, we considered we had a new prostate stem-like cell line that was named LN-NE. To evaluate the switching of cells to the neuroendocrine phenotype, we tested the expression of the neuroendocrine markers βIII-tubulin and NSE, a signature of neuroendocrine differentiation. As shown in Figure 1B, the expression of both βIII-tubulin and NSE increased from 8 days of androgen depletion compared to control parental LNCaP cells. In addition, a low expression of the androgen receptor (AR) was observed in all stages (Figure 1B), which is one important characteristic of neuroendocrine cells [3,19]. The expression of the prostate-specific membrane antigen (PSMA) progressively increased with androgen depletion, which is indicative of a more aggressive phenotype [20]. These results indicate that the neuroendocrine differentiation observed upon androgen deprivation was maintained at least up to 3 months.

To evaluate whether the neuroendocrine phenotype was linked to the induction of stemness properties and plasticity in prostate cancer, we determined the expression of the well-known stem markers CD133 and ALDH1A1. The pentaspan transmembrane glycoprotein CD133 is the most frequently used cell surface antigen to detect CSCs from various solid tumors including prostate tumors [21] and to isolate prostate stem cells from a population of primary human prostate cancer cell lines [22]. In addition, it is overexpressed in aggressive androgen-independent prostate cancer [23]. The enzyme ALDH1A1 has also been considered a cancer stem marker in prostate cancer [24]. Neither parental nor 8 days androgen-depleted cells expressed CD133 or ALDH1A1 (Figure 1C). However, from 30 days, an increase in the expression of ALDH1A1 can be observed, and at 90 days, both the expressions of CD133 and ALDH1A1 were markedly increased (Figure 1C). To confirm the transdifferentiation of cells to stem-like cells at 90 days, we analyzed by qPCR the expression of the transcription factors Nanog and Oct4, which are master regulators of pluripotency, self-renewal, and maintenance of stem cells [25]. As shown in Figure 1D, at 90 days of androgen deprivation, the expressions of Nanog and Oct4 were remarkably enhanced. In addition, the expression of the efflux transporter ABCB1A (or P-glycoprotein), which is involved in multidrug resistance, was notably up-regulated (Figure 1D). 

We wonder whether other strategies to deplete androgen signals also induced neurodifferentiation and/or stem-like properties. Hence, we adapted LNCaP cells to grow in the presence of the AR antagonist hydroxyflutamide (FLU), and at two months cells were resistant to FLU and named LN-FLU. Then, we analyzed the expression of the neuroendocrine as well as of the stem cell markers. Figure 1B shows that LN-FLU increased the expression of NSE, indicating that these cells had developed a neuroendocrine phenotype. Moreover, the expressions of the stem cell markers CD133, ALDH1A1, ABCB1A, as well as that of the transcription factors Nanog and Oct4, were increased in LN-FLU cells (Figure 1C,D).

To address whether stemness was generally associated with neuroendocrine differentiation, we determined stem markers in the cell line LNCaP-abl, an androgen-independent cell line directly derived from LNCaP by maintaining in an androgen-depleted medium [26]. Interestingly, although as expected, LNCaP-abl cells had increased expression of the neuroendocrine markers βIII-tubulin and NSE, they did not show enhanced expression of the stem cell markers, which remained at levels comparable to parental LNCaP cells (Figure 1C,D). It is worthy to note that, as previously described [26], the expression of androgen receptor in LNCaP-abl cells compared to that of LNCaP cells, was not decreased as occurred in LN-NE and LN-FLU cells (Figure 1B).

**Figure 2:**
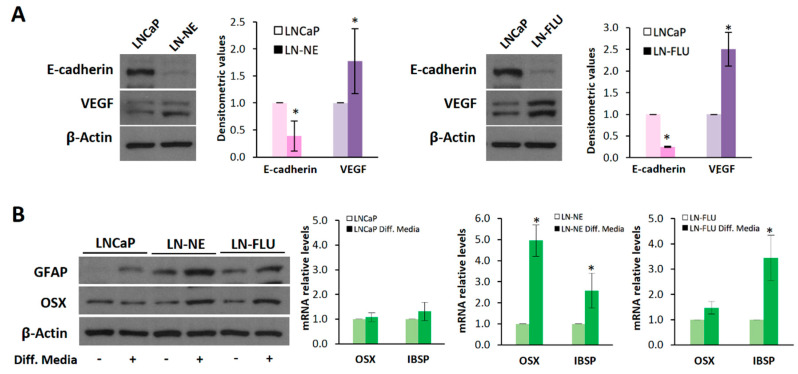
The prostate cell lines LN-NE and LN-FLU display phenotypic features of stem cells. (**A**) Levels of the epithelial marker E-cadherin and the proangiogenic factor VEGF in androgen-depleted cell lines determined by Western blot and limited to actin as a load control. A representative image of three experiments is shown. Densitometric analysis (n = 3) is shown on the right. (**B**) LNCaP, LN-NE, and LN-FLU cells were incubated in differentiation media for 15 days, and levels of the glial marker GFAP or the osteoblast marker Osx were determined by Western blot. Levels of expression of the osteoblast markers OSX and IBSP determined by qPCR. The data show the relative mRNA expression to GAPDH, which was used as a housekeeping gene. Data represent the mean ± SD of two independent experiments. * *p* < 0.05 significant difference between LNCaP and LN-NE cells or LNCaP and LN-FLU cells by two-way ANOVA and Sidak’s multiple comparisons test.

We then investigated the effects of androgen withdrawal on phenotypic features of stem cells. The epithelial–mesenchymal transition (EMT) plays a pivotal role in stem cell differentiation [27,28]. Loss of epithelial tight cell–cell junctions is accompanied by a decrease in the expression of E-cadherin, which is considered a key event in an EMT. To further corroborate the stemness acquisition of the prostate cells, E-cadherin expression was analyzed by Western blot. As expected, long-term androgen withdrawal induced a decrease in E-cadherin in LN-NE and LN-FLU cells confirming the loss of epithelial characteristics (Figure 2A). Another feature of cancer stem cells is the angiogenesis activation by releasing the vascular endothelial growth factor (VEGF). Additionally, autocrine VEGF signaling can promote an EMT phenotype, and these two phenomena may function in concert to promote CSC properties [29]. As shown in Figure 2A, the expression of VEGF was increased both in LN-NE and LN-FLU cells compared to their parental cells, additionally demonstrating the acquisition of stem-like properties.

We further evaluated whether the prostate stem cells were able to reprogram to the other cell lineage, a typical feature of stem cells. To assess the re-programming capability of the prostate stem cells, we incubated the cells for two weeks in the presence of glial or osteocyte conditioned media to induce differentiation towards astrocytes or osteoblasts, two cell lineages very different to epithelial prostate cells. Figure 2B shows that medium supplementation with osteoblastic differentiation factors promoted the expression of Osterix (Osx), a zinc-finger-family transcriptional factor essential for osteoblast differentiation, and that of the canonical osteoblast marker bone sialoprotein (IBSP), in the LN-NE and LN-FLU stem-like cells, compared to the parental LNCaP cells. Likewise, the expression of the glial fibrillary acidic protein (GFAP), an intermediate filament protein highly expressed in astrocytes, increased in LN-NE and LN-FLU cells when incubated in glial differentiation media, while it did not change in the parental cells (Figure 2B). These results indicate that LN-NE and LN-FLU cells present the ability to reprogram into different cell lineages, a hallmark feature of stem cells.

### 3.2. Prostate Stem-Like Cells are Resistant to Docetaxel and 2-Hydroxyflutamide

**Figure 3:**
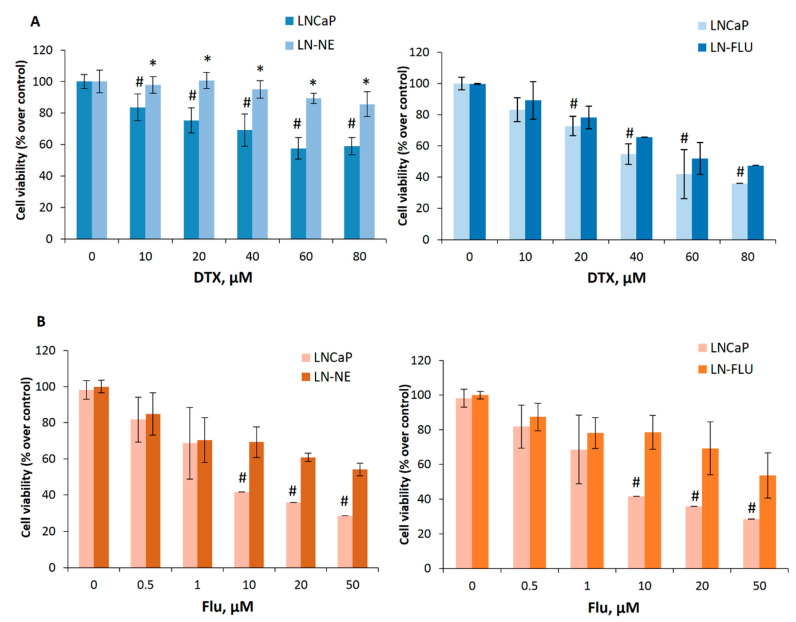
The cell lines LN-NE and LN-FLU are resistant to docetaxel and to 2-hydroxyflutamide. (**A**) LNCaP, LN-NE, and LN-FLU cells were incubated with increasing concentrations of docetaxel (DTX) for 24 h. Cell viabilities were determined by MTT assay and expressed as percentages of control (DMSO treatment). Experiments were run in quadruplicate and carried out at least two times on separate occasions. (**B**) LNCaP, LN-NE, and LN-FLU cells were incubated with increasing concentrations of 2-hydroxyflutamide (FLU) for 24 h. Cell viabilities were determined by MTT assay and expressed as percentages of control (DMSO treatment). Experiments were run in quadruplicate and carried out at least two times on separate occasions. *p < 0.01 significant difference between stem-like and parental cells and # *p* < 0.01 between treated and non-treated cells by two-way ANOVA and Tukey’s multiple comparisons test.

We then aimed to investigate the sensitivity of the new cell lines LN-NE and LN-FLU to conventional chemotherapy. To that end, we treated cells with increasing concentrations of the taxane docetaxel and analyzed cell viability by MTT. Whereas the viability of parental LNCaP cells decreased with docetaxel, docetaxel was less effective in LN-NE and LN-FLU cells (Figure 3A). Then, we tested the effect of the antiandrogen 2-hydroxyflutamide on prostate cell viability. We found that LN-NE cells were less sensitive to the antiproliferative effect of the antiandrogen, and, as expected, LN-FLU cells were resistant to 2-hydroxyflutamide (Figure 3B). This indicates that the new prostate stem-like cells were resistant to different chemotherapies, independently of the drug used to induce resistance.

### 3.3. Reprogramming to Stem-Like Cells is Associated with a Decrease in AMPK Expression

**Figure 4:**
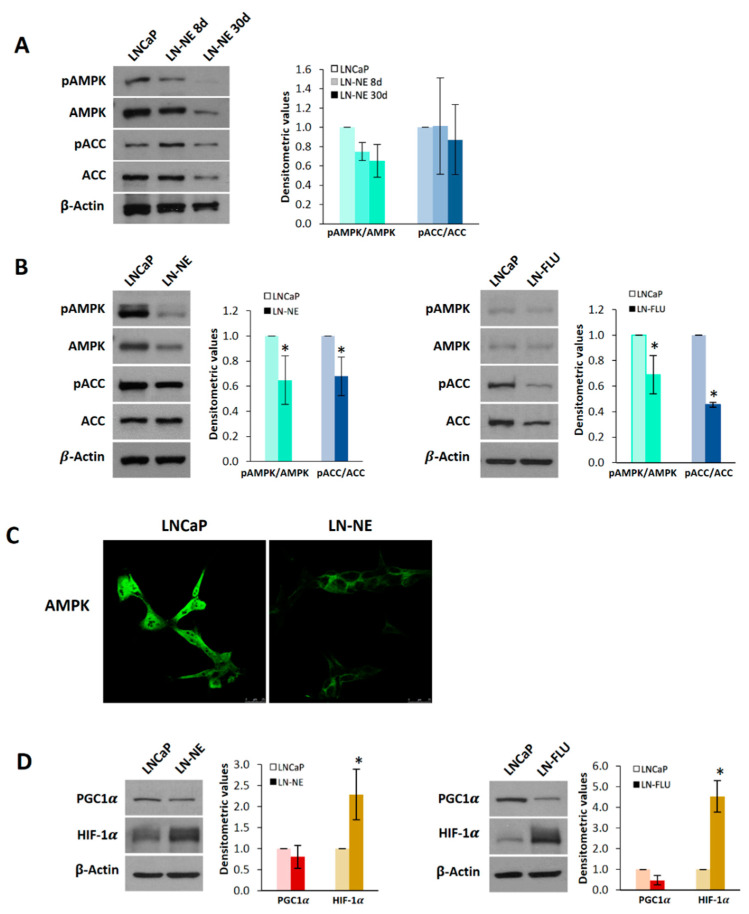
AMP-activated kinase (AMPK) is down-regulated in prostate stem-like cells. (**A**) Levels of phosphorylated AMPK (pAMPK), total AMPK, phosphorylated ACC (pACC), and total ACC were determined in LN-NE cells at 8 and 30 days by Western blot. Image is representative of three different experiments. Densitometric values are shown on the right. (**B**) Levels of pAMPK, AMPK, pACC, and ACC in LN-NE cells at 90 days of androgen withdrawal and in LN-FLU cells at two months. Image is representative of four different experiments. Densitometric values are shown on the right of each image. (**C**) Confocal images showing AMPK immunostaining in LN-NE cells at 90 days. (**D**) Levels of the transcriptional coactivator PGC-1α and the hypoxia-inducible factor HIF-1α in LN-NE and LN-FLU cells determined by Western blot. Image is representative of three different experiments. Densitometric values are shown on the right of each image. * *p* < 0.001 significant difference between LNCaP and LN-NE cells or LNCaP and LN-FLU cells by two-way ANOVA and Sidak’s multiple comparisons test.

We have recently demonstrated that activation of AMPK sensitizes PC3 prostate cancer cells to docetaxel [30]; therefore, we aimed to explore the expression pattern and activity of AMPK in docetaxel-androgen-resistant LN-NE cells. As shown in Figure 4A, the levels and phosphorylation of AMPK alpha subunit in Thr 172, which is indicative of its activation, progressively decreased upon androgen withdrawal. At 90 days, AMPK phosphorylation was notably decreased in LN-NE cells (Figure 4B). To corroborate this result, we measured the phosphorylation in Ser79 of the well-known downstream AMPK substrate, acetyl CoA carboxylase (ACC), which was also reduced (Figure 4B) further confirming the diminution of AMPK activity. The downregulation of AMPK was additionally supported by confocal microscopy (Figure 4C).

To mechanistically analyze the stemness induction of LN-NE cells, we investigated the expression of peroxisome proliferator-activated receptor gamma coactivator-1 alpha (PGC-1α), a downstream AMPK target and a critical regulator of cell differentiation [31,32]. The analysis of PGC1α levels revealed that this coactivator was downregulated in stem-like LN-NE and LN-FLU cells (Figure 4D), in concordance with AMPK levels in those cells. This result suggests a less differentiated state of LN-NE cells, which is in good agreement with their stemness. The hypoxia-inducible factor 1α (HIF-1α) has been involved in the proliferation, stemness, and reprograming of stem cells [33]. Activation of mammalian target of rapamycin (mTOR) and loss of tumor suppressors like LKB1 may activate HIF-1 by enhancing HIF-1α protein synthesis. AMPK inactivates mTOR signaling and, hence, inhibits HIF-1α expression. Since AMPK was down-regulated in the prostate stem-like LN-NE and LN-FLU cells, we wondered whether HIF-1α would be up-regulated. As expected, Figure 4D shows that HIF-1α was increased in LN-NE as well as in LN-FLU cells, providing a potential mechanism linking AMPK depletion and stemness. 

**Figure 5:**
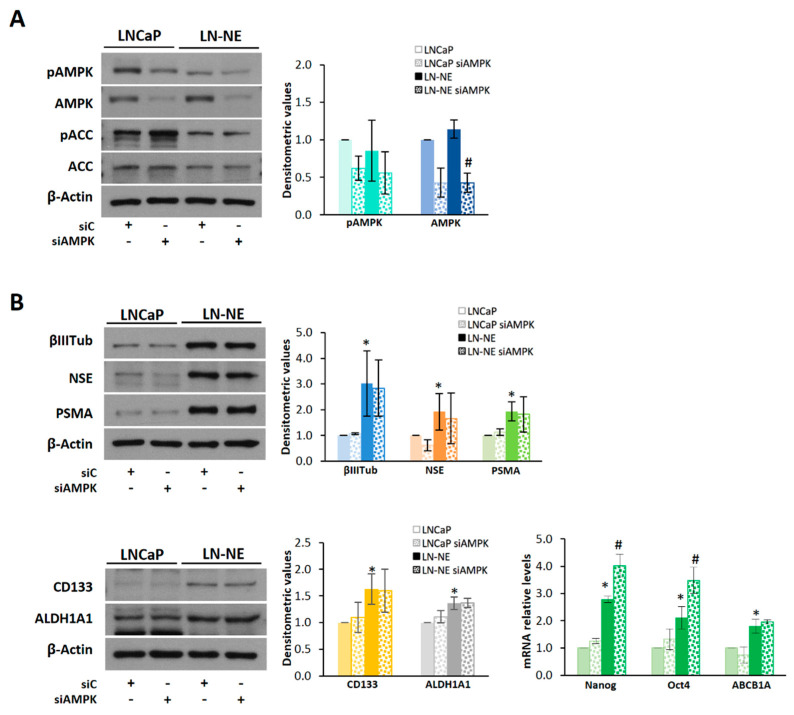
Knocking-down of AMPK in LN-NE cells increases the expression of Nanog and Oct4. (**A**) AMPK expression was downregulated by siRNA in LN-NE cells, and levels of the AMPK/ACC pathway proteins were determined by Western blot. (**B**) Levels of neuroendocrine and stem markers in AMPK knocked-down cells determined by Western blot and by qPCR. Densitometric values are shown on the right of each image. *p < 0.01 significant difference between LN-NE and LNCaP cells by two-way ANOVA and Sidak’s multiple comparisons test, # *p* < 0.05 significant difference between siAMPK and siC cells.

To address the impact of AMPK depletion on the expression of stem markers, we knocked-down AMPK by siRNA (Figure 5A). AMPK knockdown induced a higher expression of the stem markers Nanog and Oct4 both in LN-NE cells and in the parental LNCaP cells (Figure 5B).

**Figure 6:**
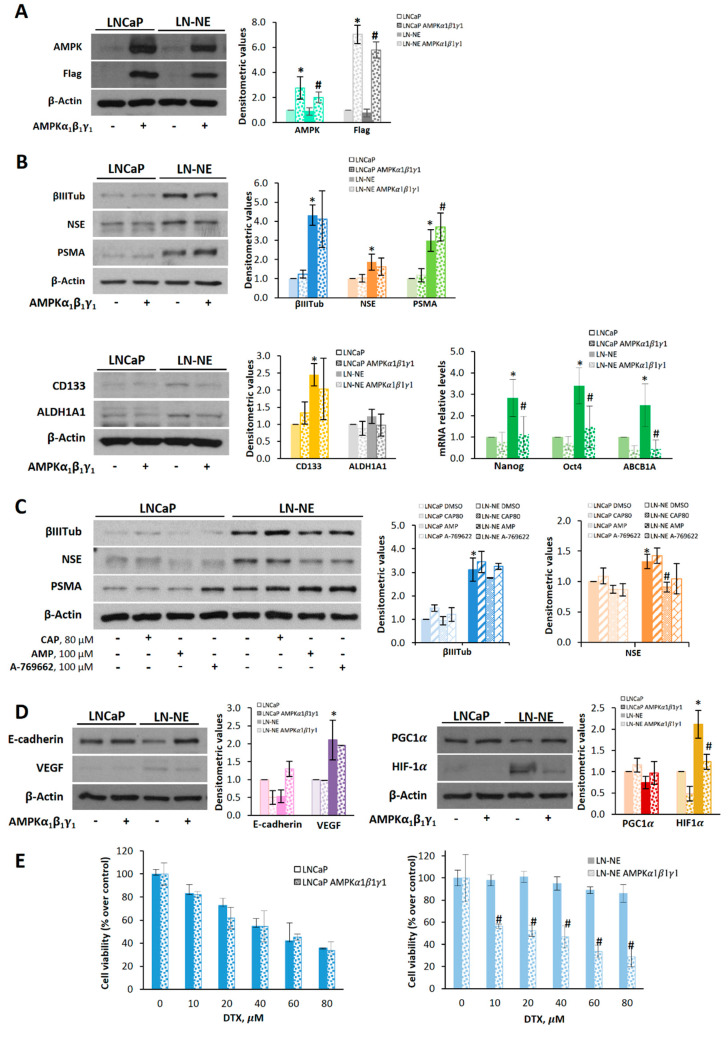
AMPK overexpression or AMPK activation decreases neuroendocrine and stem markers in LN-NE cells and restores sensitivity to docetaxel. (**A**) AMPKα1β1γ1 was overexpressed in LN-NE cells by transfection with Flag-tagged plasmids containing the three AMPK subunits. (**B**) Effect of the overexpression of AMPKα1β1γ1 on neuroendocrine markers and on CD133 and ALDH1A1 determined by Western blot. Images are representative of three independent experiments. Densitometric analysis of bands is shown. Levels of Nanog, Oct4, and ABCB1A were determined by qPCR. (**C**) LN-NE cells were treated with capsaicin (CAP), AMP, or A-769662 for 24 h, and levels neuroendocrine markers were determined by Western blot. (**D**) Levels of E-cadherin and VEGF as well as levels of PGC-1α and HIF-1α in AMPK transfected cells were determined by Western blot. Image is representative of two different experiments. Densitometric values are shown on the right of each image. (**E**) Effect of the transient expression of AMPKα1β1γ1 on cell viability in LNCaP and LN-NE cells treated with increasing concentrations of DTX. Experiments were run in quadruplicate and carried out two times on separate occasions (data are the mean ± SD). * *p* < 0.01 significant difference between LN-NE and LNCaP cells by two-way ANOVA and Sidak’s multiple comparisons test, # *p* < 0.01 significant difference between AMPK-transfected and non-transfected cells.

Interestingly, overexpression of AMPK by transient transfection (Figure 6A) reduced, although not significantly, the expression of neuroendocrine markers CD133 and ALDH1A1 in LN-NE cells (Figure 6B). In addition, the expressions of Nanog, Oct 4, and ABCB1A significantly decreased in the LN-NE stem-like cells transfected with AMPK (Figure 6B). Other authors have recently demonstrated that activation of AMPK with the thienopyridone A-769662 inhibited the reprogramming of mouse embryonic and human diploid fibroblasts into stem cells [14]. Accordingly, and to corroborate the involvement of AMPK on neuroendocrine markers, we activated AMPK pharmacologically. To that end, we used the natural compound capsaicin, which we have shown to indirectly activate AMPK in prostate cells [30,34], as well as AMP and A-769662, which are allosteric AMPK activators. As shown in Figure 6C, AMP and A-769662 reduced the levels of βIII-tubulin and NSE in LN-NE cells.

We then investigated whether AMPK transfection impacted epithelial–mesenchymal transition and angiogenesis. As shown in Figure 6D, LN-NE stem-like cells transfected with AMPK restored E-cadherin levels and decreased the expression of VEGF. Likewise, PGC-1α and HIF-1α levels in stem-like transfected cells were comparable to those of the parental cells, further supporting the role of AMPK on stemness reversion and cell reprogramming. 

Finally, we analyzed whether AMPK overexpression had an effect on the drug sensitivity of prostate LN-NE stem-like cells. As shown in Figure 6E, docetaxel inhibited cell viability in AMPK-transfected stem-like cells, whereas it had no effect on LN-NE cells. 

All these findings indicate that AMPK can induce prostate stem cell reprogramming and restore docetaxel sensitivity.

## 4. Discussion

**Figure 7:**
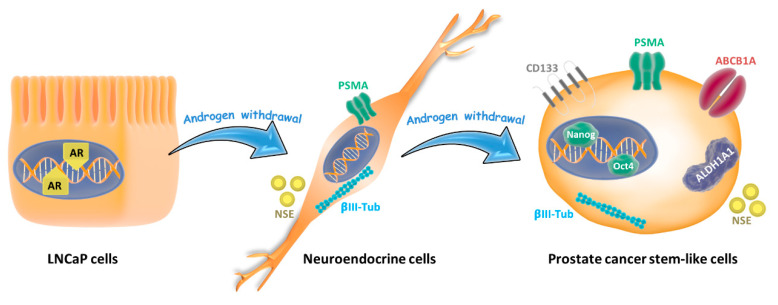
Lineage plasticity in prostate tumor cells induced by androgen depletion. Androgen-sensitive prostate cancer cells (LNCaP cells) grown in androgen withdrawal undergo a phenotype switching and exhibit neuroendocrine features like neurite outgrowth and expression of neuron-specific enolase (NSE) and βIII-tubulin. When cells are maintained for more than 30 days in androgen withdrawal, they re-differentiate to stem-like cells, increasing the expressions of CD133, ALDH1A1, and ABCB1A proteins, which are related to drug resistance, and the pluripotent factors Nanog and Oct 4.

Tumor cell conversion into a different phenotype has been associated with therapeutic resistance [4]. Approximately 20% of advanced prostate cancer patients experience cell plasticity and the acquisition of new phenotypes often associated with loss of androgen receptor signaling [35]. In this study, we demonstrated that androgen signal depletion by charcoal stripping of FBS or by the AR antagonist 2-hydroxyflutamide induced a neuroendocrine phenotype followed by re-differentiation towards an alternative “stem-like” state (Figure 7) that was resistant to chemotherapy. Those drug-resistant stem-like cells, compared to the parental LNCaP cells, displayed characteristic up-regulation of CD133, ALDH1A1, ABCB1, and the transcription factors Nanog and Oct4. This model resembles the behavior of some prostate tumors to AR-targeted therapies. 

Charcoal is used to remove androgens and estrogens from FBS to produce androgen-free media when this charcoal-stripped FBS is added to growth media instead of FBS. This model has been used to generate many androgen-suppressed LNCaP sublines. For instance, LNCaP cells grown in charcoal-stripped media for more than one year grew slowly, had very low levels of androgen receptor, displayed a neuroendocrine-like morphology, and were resistant to apoptosis-inducing agents [36]. However, authors did not evaluate the expression of stem markers or stem features. The subline LNCaP 104-S progressed to a slow growing stage (104-R1) and then to a faster growing stage (104-R2) during more than 2 years of continuous culture in the absence of androgen [37]. Those cells expressed high levels of androgen receptor and were sensitive to androgens, which induced cell growth that was blocked by antiandrogens [37]. Curiously, restoration of testosterone levels could suppress the growth of LNCaP 104-R2 hormone-refractory prostate tumors [38], indicating that an intermittent ADT could be more effective for very long treatments. Lu et al. developed a LNCaP cell subline capable of growing in charcoal-stripped serum (LNCaP-AI), which expressed a similar level of androgen receptor than their parental cells and were sensitive to androgen stimulation [39]. After three weeks of culture in charcoal-stripped serum, the majority of the cells showed neuroendocrine-like phenotype [39]. Culig et al. established a new LNCaP-abl subline by propagation for ten months in androgen-depleted medium [26]. LNCaP-abl cells grew on clusters, had a high expression and activity of the androgen receptor, and were inhibited by the anti-androgen bicalutamide [26]. LNCaP-abl cells have neuroendocrine morphology and express high levels of neuroendocrine markers [40]. In a more recent study, Wang et al. demonstrated that LNCaP spheres grown in serum-free media for 12 days had enhanced expression of the membrane stem markers CD24 and CD44 and were resistant to apoptotic stimulus [41]. Most of these cell lines displayed neuroendocrine morphology and were resistant to toxic stimuli. However, the sensitivity to androgens as well as the level of androgen receptor expression varies between them, being higher in longer incubation periods. Unfortunately, stem markers or stem features were not determined in most of the LNCaP sublines. We incubated LNCaP cells in charcoal-stripped medium for 90 days. Although we have not evaluated androgen sensitivity, cells had low levels of AR and were resistant to docetaxel and 2-hydroxyflutamide. After three months of growing in an androgen-depleted medium, LN-NE cells showed a high expression of stem markers and exhibited stem features. Interestingly, neuroendocrine markers were also increased in LNCaP cells adapted to grow in the presence of 2-hydroxyflutamide showing a new strategy to induce neuroendocrine differentiation. By contrast, the LNCaP-abl subline, which showed high levels of AR, did not enhance the expression of stem markers, suggesting an involvement of AR in stemness that will be researched in a future work.

Prostate tumors most frequently display epithelial adenocarcinoma morphology, but around one-quarter of resistant prostate tumors may reprogram toward alternative pathways adopting features of neuroendocrine cells [42] or other cell lineages [43]. Even though this phenomenon represents a challenging barrier in clinical management of patients, as there are no established therapeutic approaches for this kind of tumor [35], research in this field has not been as intensive as desirable, and mechanisms underlying transdifferentiation remain poorly defined. Our model can help to study molecular and morphological characterizations involved in prostate plasticity induced by therapy. It is worthy to note that, though cell reprogramming was induced by androgen withdrawal, cell lines generated were resistant to docetaxel as well. The increase of ALDH1A1 and ABCB1A observed in LN-NE and LN-FLU cell lines may explain therapy resistance, as these proteins are critical in detoxifying and extrusion processes, respectively. As previously described, docetaxel has high affinity for the transporter ABCB1A, which results in a decrease in the intracellular concentration of this agent in cancer cells, and ABCB1A inhibition is sufficient to overcome docetaxel resistance [44,45]. Furthermore, recent research has demonstrated that CD133 is also implicated in drug resistance since CD133 suppression enhanced the chemosensitivity of prostate cells to paclitaxel and decreased cell proliferation, migration, and invasion [46]. Furthermore, E-cadherin expression was decreased in LN-NE and LN-FLU stem-like cells suggesting epithelial–mesenchymal transition, which additionally may contribute to chemoresistance in cancer.

Our study shows that prostate phenotype switching was accompanied by a decrease in AMPK expression and activation. Interestingly, AMPK pharmacological activators or AMPK transfection reduced neuroendocrine and stem cell markers reinforcing the involvement of AMPK on prostate cell plasticity. Moreover, our results show that AMPK overexpression in stem-like cells restored E-cadherin, VEGF, PGC-1α, and HIF-1 α levels, further demonstrating a role for AMPK in the regulation of stemness. In line with our results, it has been demonstrated that the indirect and direct activation of AMPK with the antidiabetic biguanide metformin and the compound A-769662, respectively, impeded the reprogramming of mouse embryonic and human diploid fibroblasts into pluripotent stem cells [14]. In addition, results by Wang et al. demonstrated a regulatory role of AMPK in prostate cancer stemness. AMPK triggered Nanog degradation by blocking B-Raf-induced stabilizing phosphorylation of Nanog leading to inhibition of cell reprogramming [47], which is in good agreement with our results. Further research has evidenced that the AMPK indirect activator metformin impairs cancer stem cells by targeting of specific pathways involved in cell differentiation, renewal, metastasis, and metabolism [48]. Metformin significantly inhibits the sphere-forming ability of breast, pancreatic, and ovarian CSCs, and in combination with chemotherapeutic drugs it markedly reduces the CSC subpopulation and tumor volume [29]. Though metformin has demonstrated an overall benefit in prostate cancer risk [49,50] and a synergic antitumoral effect in combination with other drugs and radiation therapy [51,52], metformin’s effect on prostate cancer stem cells remains to be elucidated [53]. On the other hand, it has been shown that AMPK may play a role in morphogenesis. For instance, AMPK activation after osmotic stress in vitro can cause loss of potency factors such as Cdx2 and Id2, thereby influencing cellular differentiation [54]. Hence, AMPK may be relevant in regulating cell fate and plasticity [55]. Even so, the underlying molecular mechanism whereby AMPK regulates cell plasticity remains elusive. In agreement with our results, it has been documented that AMPK activation by metformin inhibits angiogenesis by suppression of HIF-1α and VEGF [56]. Moreover, disruption of AMPK signaling, either though inhibition of AMPK itself or its upstream kinase LKB1, induces elevated HIF-1α protein expression and a pro-growth metabolic reprogramming in cancer cells [57,58]. It has been demonstrated that, in prostate cells, HIF-1α leads to increased expression of VEGF, IL-6, and CSC marker genes such as Nanog, Oct4, and EZH2 [59], which is in good concordance with our results. Most importantly, in our study we found that AMPK overexpression could overcome chemoresistance, increasing cell sensitivity to docetaxel. These findings demonstrate that AMPK can decrease the stemness phenotype of prostate cancer cells to induce drug sensitivity. 

Through the implication of AMPK in prostate cell reprogramming, our work provides a new regulatory mechanism of prostate cancer stemness. Therefore, targeting AMPK could overcome mechanisms involved in the acquisition of drug resistance and might be a promising and efficient therapeutic strategy for patients with prostate cancer. This system will facilitate development of novel therapeutic agents and allow further exploration into mechanisms governing the transformation to hormone refractory prostate cancer.
